# Effects of coordinating heteroatoms on molecular structure, thermodynamic stability and redox behavior of uranyl(vi) complexes with pentadentate Schiff-base ligands†

**DOI:** 10.1039/d2ra04639c

**Published:** 2022-08-26

**Authors:** Tomoyuki Takeyama, Koichiro Takao

**Affiliations:** Laboratory for Zero-Carbon Energy, Institute of Innovative Research, Tokyo Institute of Technology 2-12-1 N1-32, O-Okayama, Meguro-ku Tokyo 152-8550 Japan takeyama.t.ab@m.titech.ac.jp takao.k.ac@m.titech.ac.jp

## Abstract

Uranyl(vi) complexes with pentadentate N_3_O_2_-, N_2_O_3_- and N_2_O_2_S_1_-donating Schiff base ligands, *t*Bu,MeO–saldien–X^2−^ (X = NH, O and S), were synthesized and thoroughly characterized by ^1^H NMR, IR, elemental analysis, and single crystal X-ray diffraction. The crystal structures of UO_2_(*t*Bu,MeO–saldien–X) showed that the U–X bond strength follows U–O ≈ U–NH > U–S. Conditional stability constants (*β*_X_) of UO_2_(*t*Bu,MeO–saldien–X) in ethanol were investigated to understand the effect of X on thermodynamic stability. The log *β*_X_ decrease in the order of UO_2_(*t*Bu,MeO–saldien–NH) (log *β*_NH_ = 10) > UO_2_(*t*Bu,MeO–saldien–O) (log *β*_O_ = 7.24) > UO_2_(*t*Bu,MeO–saldien–S) (log *β*_S_ = 5.2). This trend cannot be explained only by Pearson's Hard and Soft Acids and Bases (HSAB) principle, but rather follows the order of basicity of X. Theoretical calculations of UO_2_(*t*Bu,MeO–saldien–X) suggested that the ionic character of U–X bonds decreases in the order of U–NH > U–O > U–S, while the covalency increases in the order U–O < U–NH < U–S. Redox potentials of all UO_2_(*t*Bu,MeO–saldien–X) in DMSO were similar to each other regardless of the difference in X. Spectroelectrochemical measurements and DFT calculations revealed that the center U^6+^ of each UO_2_(*t*Bu,MeO–saldien–X) undergoes one-electron reduction to afford the corresponding uranyl(v) complex. Consequently, the difference in X of UO_2_(*t*Bu,MeO–saldien–X) affects the coordination of *t*Bu,MeO–saldien–X^2−^ with UO_2_^2+^. However, the HSAB principle is not always prominent, but the Lewis basicity and balance between ionic and covalent characters of the U–X interactions are more relevant to determine the bond strengths.

## Abstract 2

The U–X bond strength and thermodynamic stability of uranyl(vi) complexes with pentadentate N_2_O_2_X_1_-donating ligands (X = NH, O, S) are affected by the difference in X. In contrast, the X atom does not largely affect the redox behavior of the complexes.

## Graphical abstract

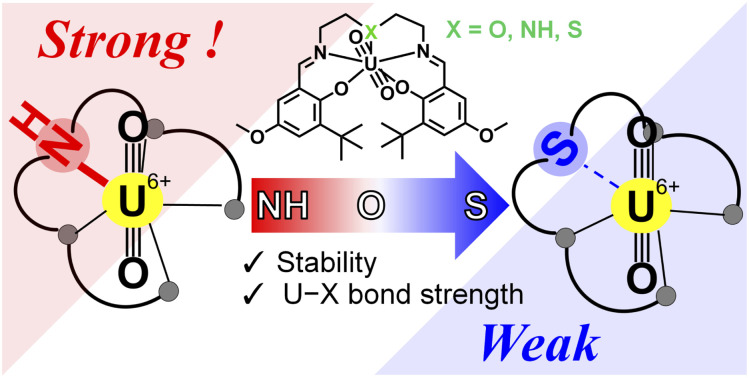

## Introduction

Uranium is the most important element in nuclear engineering. The chemistry of uranium plays important roles in nuclear fuel fabrication and spent fuel reprocessing. Under ambient conditions, uranium is most commonly present as a hexavalent uranyl(vi) ion, UO_2_^2+^, with a typical linear [O≡U^VI^≡O]^2+^ structure. The chemical separation of UO_2_^2+^ from various aqueous systems such as feed solutions of spent nuclear fuels and even seawater is one of the important research topics in nuclear chemistry. In the usual sense, coordination chemistry provides very powerful tools for chemical separation. Hence, the complexation between UO_2_^2+^ and organic ligands has been widely studied.^1–13^

Pearson's Hard and Soft Acids and Bases (HSAB) principle is quite useful to describe preferential interactions between specific metal ions and coordinating atoms of ligands and to design organic molecules selectively coordinating with a target metal ion,^14,15^ although this principle is rather empirical. In the HSAB principle, UO_2_^2+^ is classified as a hard acid,^14,15^ and therefore, generally tends to more strongly interact with hard bases like N, O and F, compared with softer ones such as heavier congeners like P, S, and Cl.^14,15^ Indeed, thermodynamic stability of a UO_2_^2+^-halido complex in DMF follows the order of hardness of halide ligands, Cl^−^ > Br^−^ > I^−^.^16^ In contrast, such a trend in complexation between UO_2_^2+^ and heteroatoms like N, O and S, seems not to be well understood systematically, although it would provide essential information to understand the fundamental nature of UO_2_^2+^ in more depth and to design molecular structures of ligands exclusively interacting with UO_2_^2+^. Indeed, several extracting reagents have been successfully developed for separation of Am(iii) and Cm(iii) from Ln(iii) on the basis of difference in coordinating affinities of these metal ions with soft-donor atoms incorporated in the designed ligands.^17–20^

**Fig. 1:**
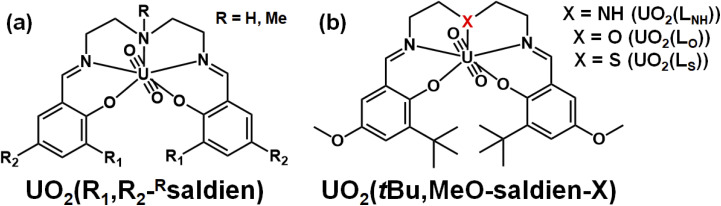
Schematic structures of UO_2_(R_1_,R_2_,–^R^saldien)^21,22^ (a) and UO_2_(*t*Bu,MeO–saldien–X) complexes (UO_2_(L_X_)); (X = NH, O, S) (b).

In this study, we discuss strengths of U–N, U–O and U–S interactions formed in UO_2_^2+^ complexes having analogous coordination geometries. For this purpose, it is first necessary to choose a suitable ligand system. Previously, we reported UO_2_^2+^ complexes with N_3_O_2_-pentadentate Schiff base ligands, UO_2_(R_1_,R_2_,–^R^saldien), shown in Fig. 1(a).^21,22^ Its NR moiety can be substituted with O or S to provide the similar UO_2_^2+^ complexes, UO_2_(*t*Bu,MeO–saldien–X) (UO_2_(L_X_), X = NH, O, S, Fig. 1(b)), where the U–O or U–S bond will be formed instead of U–NR. Here, we report synthesis and characterization of UO_2_(L_X_) (X = NH, O, S) to discuss effects of X to the U–X bond strength and thermodynamic stability as well as redox chemistry of this class of UO_2_^2+^ complexes.

## Experimental section

### Materials and syntheses

All reagents used were of reagent grade and used as received, if not specified. 3-*tert*-Butyl-5-methoxysalicylaldehyde was synthesized as reported elsewhere.^23^

#### UO_2_(*t*Bu,MeO–Saldien–NH) (UO_2_(L_NH_))

To a solution of 3-*tert*-butyl-5-methoxysalicylaldehyde (202 mg, 0.971 mmol) in ethanol (2 mL) was added 2,2′-diaminodiethylamine (52.1 μL, 0.480 mmol). This solution was heated to reflux for 10 min. UO_2_(NO_3_)_3_·6H_2_O (210 mg, 0.418 mmol) dissolved in ethanol (2 mL) was dropwise added to the solution. A red precipitate was formed within several minutes, and the suspension was stirred at 60 °C for 1 h. After cooling to room temperature, a red precipitate was collected by filtration and rinsed with methanol. Recrystallization from CH_2_Cl_2_/ethanol yielded red crystals. Yield: 176 mg (48%). This compound was characterized by ^1^H NMR, IR and elemental analysis. ^1^H NMR (399.78 MHz, CD_2_Cl_2_, *δ*/ppm *vs.* TMS): 1.71 (s, 18H, –C(C*H*_3_)_3_), 3.61 (m, 2H, C=N–C*H*_2_CH_2_–N or C=N–CH_2_C*H*_2_–N), 3.83 (s, 6H, O–C*H*_3_), 4.07 (m, 2H, C=N–C*H*_2_CH_2_–N or C=N–CH_2_C*H*_2_–N), 4.26 (m, 1H, –N*H*), 4.50 (m, 4H, C=N–C*H*_2_CH_2_–N or C=N–CH_2_C*H*_2_–N), 6.86 (d, 2H, aryl, *J*_H–H_ = 3.2 Hz), 7.32 (d, 2H, aryl, *J*_H–H_ = 3.2 Hz), 9.38 (s, 2H, N=C*H*–). ^1^H NMR (399.78 MHz, DMSO-*d*_6_, *δ*/ppm *vs.* TMS): 1.65 (s, 18H, –C(C*H*_3_)_3_), 3.40 (m, 2H, C=N–C*H*_2_CH_2_–N or C=N–CH_2_C*H*_2_–N), 3.75 (s, 6H, O–C*H*_3_), 4.02 (m, 2H, C=N–C*H*_2_CH_2_–N or C=N–CH_2_C*H*_2_–N), 4.49 (m, 4H, C=N–C*H*_2_CH_2_–N or C=N–CH_2_C*H*_2_–N), 6.51 (m, 1H, –N*H*), 7.04 (d, 2H, aryl, *J*_H–H_ = 3.2 Hz), 7.14 (d, 2H, aryl, *J*_H–H_ = 3.2 Hz), 9.57 (s, 2H, N=C*H*–). IR (ATR, cm^−1^): 858 (O≡U≡O asymmetric stretching, *ν*_3_), 1636 (C=N stretching, *ν*_C=N_). Elemental analysis (%) calcd for UO_2_(L_NH_) (C_28_H_39_N_3_O_6_U_1_): C, 44.74; H, 5.23; N, 5.59. Found: C 44.64; H, 5.29; N, 5.42. The obtained crystals were also suitable for the X-ray crystallography.

#### UO_2_(*t*Bu,MeO–Saldien–O) (UO_2_(L_O_))

To a solution of 3-*tert*-butyl-5-methoxysalicylaldehyde (50.7 mg, 0.243 mmol) in ethanol (3 mL) was added 2,2′-oxybis(ethylamine) (12.6 μL, 0.119 mmol). This solution was heated to reflux for 10 min. UO_2_(NO_3_)_3_·6H_2_O (210 mg, 0.418 mmol) dissolved in ethanol (2 mL) was dropwise added to the solution. A red precipitate was formed within several minutes, and the suspension was stirred at 60 °C for 1 h. After cooling to room temperature, a red precipitate was collected by filtration and rinsed with methanol. Recrystallization from CH_2_Cl_2_/ethanol yielded red microcrystals. Yield: 27.1 mg (30%). This compound was characterized by ^1^H NMR, IR and elemental analysis. ^1^H NMR (399.78 MHz, CD_2_Cl_2_, *δ*/ppm *vs.* TMS): 1.71 (s, 18H, –C(C*H*_3_)_3_), 3.83 (s, 6H, O–C*H*_3_), 4.59 (m, 4H, C=N–C*H*_2_CH_2_–O or C=N–CH_2_C*H*_2_–O), 4.65 (m, 4H, C=N–C*H*_2_CH_2_–O or C=N–CH_2_C*H*_2_–O), 6.91 (d, 2H, aryl, *J*_H–H_ = 3.2 Hz), 7.33 (d, 2H, aryl, *J*_H–H_ = 3.6 Hz), 9.50 (s, 2H, N=C*H*–). ^1^H NMR (399.78 MHz, DMSO-*d*_6_, *δ*/ppm *vs.* TMS): 1.64 (s, 18H, –C(C*H*_3_)_3_), 3.77 (s, 6H, O–C*H*_3_), 4.53 (t, 4H, C=N–C*H*_2_CH_2_–O or C=N–CH_2_C*H*_2_–O, *J*_H–H_ = 5.2 Hz), 4.66 (t, 4H, C=N–C*H*_2_CH_2_–O or C=N–CH_2_C*H*_2_–O, *J*_H–H_ = 5.2 Hz), 7.09 (d, 2H, aryl, *J*_H–H_ = 3.2 Hz), 7.17 (d, 2H, aryl, *J*_H–H_ = 3.6 Hz), 9.75 (s, 2H, N=C*H*-). IR (ATR, cm^−1^): 883 (O≡U≡O asymmetric stretching, *ν*_3_), 1637 (C=N stretching, *ν*_C=N_). Elemental analysis (%) calcd for UO_2_(L_O_) (C_28_H_38_N_2_O_7_U_1_): C, 44.68; H, 5.09; N, 3.72. Found: C 44.79; H, 5.21; N, 3.65. The crystals suitable for X-ray diffraction were obtained by recrystallization from pyridine/hexane.

#### UO_2_(*t*Bu,MeO–Saldien–S) (UO_2_(L_S_))

To a solution of 3-*tert*-butyl-5-methoxysalicylaldehyde (54.4 mg, 0.261 mmol) in ethanol (3 mL) was added 2,2′-thiobis(ethylamine) (14.5 μL, 0.126 mmol). This solution was heated to reflux for 10 min under Ar. UO_2_(NO_3_)_3_·6H_2_O (210 mg, 0.418 mmol) dissolved in ethanol (2 mL) was dropwise added to the solution under Ar. A dark red precipitate was formed within several minutes, and the suspension was stirred at 60 °C for 1 h. After cooling to room temperature, a red precipitate was collected by filtration quickly, and rinsed with deoxygenated ethanol. Recrystallization from CH_2_Cl_2_/ethanol yielded dark red plate crystals. Yield: 7.8 mg (8%). This compound was characterized by ^1^H NMR, IR and elemental analysis. ^1^H NMR (399.78 MHz, CD_2_Cl_2_, *δ*/ppm *vs.* TMS): 1.70 (s, 18H, –C(C*H*_3_)_3_), 3.74 (br, 4H, C=N–C*H*_2_CH_2_–S or C=N–CH_2_C*H*_2_–S), 3.83 (s, 6H, O–C*H*_3_), 3.74 (s, 4H, C=N–C*H*_2_CH_2_–S or C=N–CH_2_C*H*_2_–S), 5.33 (s, 1H, CH_2_Cl_2_), 6.89 (d, 2H, Aryl, *J*_H–H_ = 3.2 Hz), 7.33 (d, 2H, aryl, *J*_H–H_ = 3.6 Hz), 9.47 (s, 2H, N=C*H*–). ^1^H NMR (399.78 MHz, DMSO-*d*_6_, *δ*/ppm *vs.* TMS): 1.64 (s, 18H, –C(C*H*_3_)_3_), 3.77 (s, 6H, O–C*H*_3_), 3.79 (br, 4H, C=N–C*H*_2_CH_2_–S or C=N–CH_2_C*H*_2_–S), 4.60 (br, 4H, C=N–C*H*_2_CH_2_–S or C=N–CH_2_C*H*_2_–S), 7.09 (d, 2H, aryl, *J*_H–H_ = 3.2 Hz), 7.18 (d, 2H, aryl, *J*_H–H_ = 2.8 Hz), 9.74 (s, 2H, N=C*H*–). IR (ATR, cm^−1^): 880 (O≡U≡O asymmetric stretching, *ν*_3_), 1623 (C=N stretching, *ν*_C=N_). Elemental analysis (%) calcd for UO_2_(L_S_)·0.5CH_2_Cl_2_ (C_28_H_38_N_2_O_6_S_1_U_1_·0.5CH_2_Cl_2_): C, 42.20; H, 4.85; N, 3.45. Found: C 42.24; H, 4.79; N, 3.40. The obtained crystals were also suitable for the X-ray crystallography.

### Methods

The ^1^H NMR spectra were recorded by using JEOL ECX-400 (^1^H: 399.78 MHz) NMR spectrometer. The chemical shifts of ^1^H NMR were referenced to TMS (*δ* = 0 ppm). The IR measurements were performed by JASCO FT/IR4700 equipped with a diamond ATR attachment. Elemental analyses were carried out by Yanaco MT-6 CHN elemental analyzer. Cyclic voltammetry (CV) measurements of UO_2_(L_X_) (1 mM) dissolved in DMSO containing 0.1 M tetra-*n*-butylammonium perchlorate (TBAP) were performed at 295 K under a dry Ar atmosphere by using BAS ALS660B electrochemical analyzer. A three-electrode system consisted of a Pt disk working electrode (diameter: 1.6 mm, surface area: 0.020 cm^2^), a Pt wire counter electrode, and an Ag^0/+^ reference electrode (0.1 M TBAP + 1 mM AgNO_3_/CH_3_CN). A ferrocene/ferrocenium ion redox couple (Fc^0/+^) was taken as an external standard redox system. All samples were prepared under an inert Ar atmosphere. Dissolved oxygen gas in each sample solution was expelled by purging Ar gas for at least 10 min prior to starting the CV experiments. UV-vis-NIR spectroelectrochemical measurements in DMSO were performed with a JASCO V-770 spectrophotometer equipped with an optically transparent thin layer electrode (OTTLE) cell at 295 K.^24–27^ Its optical path length was 1.0 × 10^−2^ cm, which was calibrated spectrophotometrically.^22^ The three-electrode system was the same as that in the above electrochemical experiments with a replacement of the working electrode by a Pt gauze (80 mesh). The potential applied on OTTLE was controlled by BAS ALS660B. The absorption spectrum at each potential step was recorded after equilibration of the electrochemical reaction at the applied potential on the working electrode, which completed within 3 min. The sample solution in the OTTLE cell was prepared in a similar manner to that for the CV measurements.

### Crystallographic analysis

The X-ray diffraction data of the well-shaped single crystals of UO_2_(L_NH_)·(CH_2_Cl_2_), UO_2_(L_O_)·(C_5_H_5_N) and UO_2_(L_S_)·(CH_2_Cl_2_) were collected by a Rigaku XtaLAB mini II equipped with hybrid pixel array detector and graphite monochromated Mo Kα radiation (*λ* = 0.71073 Å). Each sample was mounted on a MiTeGen Dual Thickness MicroMounts, and located in the temperature-controlled N_2_ gas flow. Intensity data were collected by taking oscillation photographs. Reflection data were corrected for both Lorentz and polarization effects. The structures were solved by the direct method and refined anisotropically by the SHELX program suite^28^ for non-hydrogen atoms by full-matrix least-squares calculations. Each refinement was continued until all shifts were smaller than one-third of the standard deviations of the parameters involved. Hydrogen atoms were located at the calculated positions. All hydrogen atoms were constrained to ideal geometry with C–H = 0.95 Å. The thermal parameters of all hydrogen atoms were related to those of their parent atoms by *U*(H) = 1.2*U*_eq_(C). All calculations were performed by using the *Olex2* crystallographic software program package.^29^ Crystallographic data of all complexes were summarized in Table S1,† and deposited with Cambridge Crystallographic Data Centre as supplementary publication no: CCDC 2177295 (UO_2_(L_O_)·(C_5_H_5_N)), 2177296 (UO_2_(L_NH_)·(CH_2_Cl_2_)), and 2177297 (UO_2_(L_S_)·(CH_2_Cl_2_)).

### UV-vis titration

Sample solutions of H_2_L_X_ (X = NH, O, S, 0.1 mM) were prepared by mixing 3-*tert*-butyl-5-methoxysalicylaldehyde and the corresponding diamines in ethanol. The formation of H_2_L_X_ (X = NH, O, S) was checked by ^1^H NMR spectroscopy (see Fig. S1†). Triethylamine (NEt_3_, 0.4 mM) was added as a H^+^ scavenger after the formation of UO_2_(L_X_). The total concentration of UO_2_^2+^ was stepwise increased up to 0.12 mM (0 ≤ [UO_2_^2+^]/[L_X_^2−^] ≤ 1.2) by adding a feed solution of UO_2_(NO_3_)_3_·6H_2_O (10 mM) in ethanol. The UV-vis absorption spectrum at each increment step was recorded by JASCO V-770 spectrophotometer. During the whole titration experiment, temperature of the sample solution was kept at 293 K in a thermostat cell holder equipped with the spectrophotometer. The obtained titration series of the UV-vis absorption spectra was analyzed by HypSpec (version 1.1.33)^30^ to determine conditional stability constants of UO_2_(L_X_) (X = NH, O, S) under the presence of 0.4 mM NEt_3_ in ethanol.

### Theoretical calculations

Density functional theory (DFT) calculations were performed using Gaussian 16 program (Revision B.01)^31^ for characterization of UO_2_(L_X_) and the one-electron reduced complexes, [UO_2_(L_X_)]^−^ (X = NH, O, S). The atomic coordinates of UO_2_(L_X_) were taken from those experimentally-determined and were used for structure optimization. Hybrid DFT functional B3LYP^32^ was employed and solvent was modelled through a conductor-like polarized continuum model (CPCM) for DMSO (dielectric *ε* = 46.7).^33^ For uranium, Stuttgart-type small-core effective core potential (ECP) and corresponding basis set has been used.^34^ The most diffuse basis functions on uranium with the exponent 0.005 (all s, p, d, and f type functions) were omitted as in previous studies.^35–39^ The 6-311G(d) basis sets were used for other elements (C, H, N, O, S). Vibrational frequency calculations at the same level of theory confirmed that no imaginary frequency was found to be present. Single-point calculations for energetic analysis were performed using the same condition. NBO analysis were carried out by using the NBO 5.0 program.^40^ The molecular structures of [UO_2_(L_X_)]^−^ were taken from those of UO_2_(L_X_) determined experimentally and were optimized after addition of a single negative charge and doublet spin degeneracy to assume the one-electron reduction using the same condition. The Mulliken spin-density plots were illustrated by GaussView 6.1.^41^

## Results and discussion

### Synthesis and structure determination of UO_2_(L_X_) (X = NH, O, S)

Each ligand was synthesized through a condensation reaction between a 3-*tert*-butyl-5-methoxysalicylaldehyde and the corresponding diamine in ethanol, and further reacted with one equivalent of UO_2_(NO_3_)_2_·6H_2_O to afford UO_2_(L_X_). These complexes were yielded as red microcrystalline solids, which were recrystallized from appropriate solvent mixtures to obtain single crystals suitable for X-ray structure determination. The IR peaks of [O≡U≡O]^2+^ asymmetric stretching (*ν*_3_) and C=N stretching (*ν*_C=N_) of UO_2_(L_X_) were observed at around 860–880 and 1630 cm^−1^, respectively. The elemental analysis for UO_2_(L_X_) well-agreed with the expected chemical formulae of them.

**Fig. 2:**
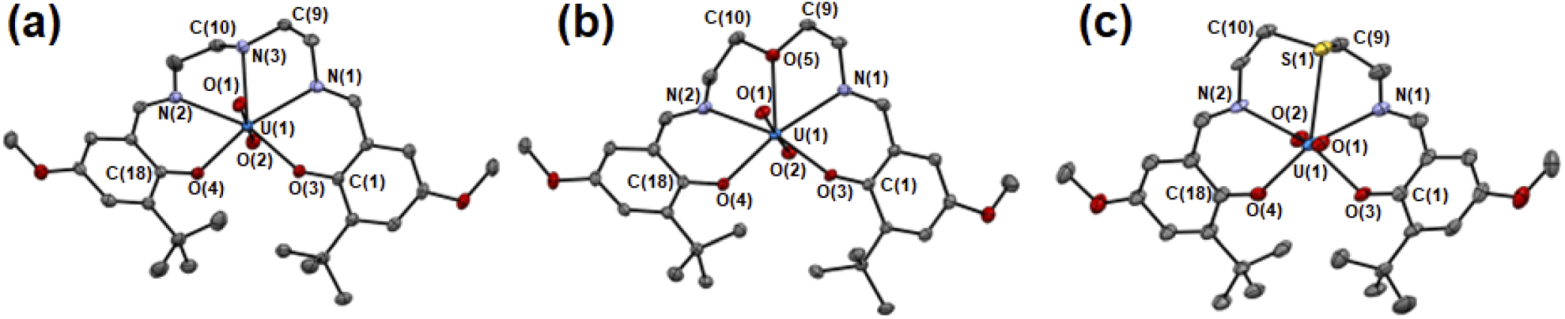
ORTEP views of UO_2_(L_NH_) (a), UO_2_(L_O_) (b), UO_2_(L_S_) (c). Ellipsoids are at 50% probability level. Hydrogen atoms and solvent molecules were omitted for clarify.

The molecular structures of UO_2_(L_X_) were determined by single crystal X-ray diffraction (SCXRD). The resulting molecular structures of UO_2_(L_X_) are shown in Fig. 2 and S2.† The selected bond lengths of them are summarized in Table 1. As a general trend, UO_2_^2+^ in UO_2_(L_X_) is five-coordinated in its equatorial plane to give a pentagonal bipyramidal coordination geometry as expected in Fig. 1(b), which is typically found in UO_2_^2+^ complexes.^21,22^

**Table 1:** Selected bond lengths (Å) of UO_2_(L_X_) (X = NH, O, S)

	UO_2_(LNH)	UO_2_(LO)	UO_2_(LS)
U–O(1)	1.793(2)	1.792(2)	1.776(5)
U–O(2)	1.787(2)	1.790(2)	1.778(5)
U–O(3)	2.238(2)	2.239(2)	2.241(6)
U–O(4)	2.239(2)	2.239(2)	2.231(5)
U–N(1)	2.535(3)	2.523(3)	2.543(7)
U–N(2)	2.554(2)	2.531(3)	2.542(6)
U–X	2.594(5)	2.581(3)	2.981(2)
C(1)–O(3)	1.342(3)	1.332(3)	1.34(1)
C(18)–O(4)	1.333(3)	1.334(3)	1.34(1)

The U≡O_ax_ bond lengths of UO_2_^2+^ in UO_2_(L_X_) (U(1)–O(1), U(1)–O(2)) are 1.78–1.79 Å, which is similar to those in UO_2_^2+^ complexes reported previously.^21,22^ Herein, we introduced *tert*-butyl groups at the *ortho*-positions of the phenolate moieties in each system to control the structure of the ligand after coordination to UO_2_^2+^. To avoid steric collision between these bulky groups in UO_2_(L_X_), two phenolate moieties are forced to be present in the opposite sides of the equatorial plane of UO_2_^2+^ (Fig. 2 and S2†). Such a twisted structure of a planar pentadentate ligand was also observed in the saldien-type ligands (*e.g.*, Fig. 1(a)) we reported previously.^21,22^

The bond angles around X are strongly affected by the difference in X. The mean bond angle of C(9)–N(3)–C(10), C(9)–N(3)–U(1) and C(10)–N(3)–U(1) in UO_2_(L_NH_) is 111°, which is close to the ideal value (109.5°) of the tetrahedral coordination around N, showing its sp^3^ character. Note that the N–H moiety in UO_2_(L_NH_) forms a hydrogen bond with the axial O of UO_2_^2+^ of the neighboring complexes (D⋯A: 3.15 Å, D–H: 1.00 Å, H⋯A: 2.210 Å, D–H⋯A: 154.9°). Nevertheless, there seems to be little effect on the structure of this UO_2_^2+^ complex, because the similar bond angles around N was also observed in UO_2_(*t*Bu,MeO–^Me^saldien) (109°, see Fig. 1). The mean bond angles around X in UO_2_(L_O_) and UO_2_(L_S_) are 120° and 98°. Moreover, the deviation of X from the mean planes defined by U1, N1, N2, O3, and O4 in UO_2_(L_S_) (0.489 Å) is larger than those of UO_2_(L_NH_) (0.261 Å) and UO_2_(L_O_) (0.116 Å). Such a difference would be related to the bonding nature and steric factor of these X atoms. However, it is difficult at this moment to clearly describe in detail how the hardness/softness of X affects such a structural trend. Hence, we decided to focus on the bond lengths around X as another structural parameter directly affected by the coordination strength.

In the UO_2_(L_X_) complexes studied here, the bond lengths between U and the phenolic O (U(1)–O(3), U(1)–O(4)) are 2.23–2.24 Å regardless of difference in X. This is also the case for those between U and the imino N (U(1)–N(1), U(1)–N(2), 2.52–2.55 Å). In contrast, the U(1)–X bond lengths depend on X. The U(1)–N(3) distance of UO_2_(L_NH_) is 2.594(5) Å, which is slightly longer than the corresponding interaction in UO_2_(L_O_) (U(1)–O(5) = 2.581(3) Å). These bond lengths are commonly found in the previous reports.^21,22^ The U(1)–S(1) distance in UO_2_(L_S_) is significantly longer than the others. However, the U(1)–S(1) distance of UO_2_(L_S_) is still shorter than the sum of van der Waals radii of U and S (2.3 Å + 1.8 Å = 4.1 Å),^42^ suggesting that chemical bonding interaction is certainly present between U(1) and S(1) in this complex. Indeed, the U(1)–S(1) distance of UO_2_(L_S_) (2.981(2) Å) is close to the U–S bond lengths in UO_2_^2+^–thioether complexes reported previously (2.96–3.02 Å).^2,43,44^ The observed structural parameters of the U–X interactions in UO_2_(L_x_) are quite common in uranyl complexes having X atom coordination reported so far.^2,21,22,43,44^ Therefore, UO_2_(L_X_) studied here are suitable for exploring impacts of X in the coordination chemistry of UO_2_^2+^.

It could be misleading to discuss the strengths of the U–X bonding interactions solely on the basis of the observed bond lengths, because the sizes of N, O and S are different from each other. The bond strengths between two atoms can be normalized by reduction in an interaction distance (*R*_UX_) derived from the sum of van der Waals radii and an actual bond length between U and X as shown in eqn (1).^45–47^

1 *R*_UX_ = (*d*_UX_)/(*r*_U_ + *r*_X_)

where *r*_U_ and *r*_X_, are van der Waals radii of U and X, respectively. *d*_UX_ is the U–X bond length of UO_2_(L_X_) determined by SCXRD.^45–47^ Based on this definition, greater *R*_UX_ implies weaker U–X bond (*vice versa*). As a result, *R*_UX_ of UO_2_(L_NH_) is 0.665, which is close to that of UO_2_(L_O_) (0.670). This implies that the bond strengths of U(1)–N(3) in UO_2_(L_NH_) and U(1)–O(5) in UO_2_(L_O_) are similar to each other. In contrast, *R*_UX_ of UO_2_(L_S_) is 0.727, which is significantly greater than those of UO_2_(L_NH_) and UO_2_(L_O_). Hence, the U(1)–S(1) bond strength of UO_2_(L_S_) is supposed to be weaker than the U–X ones in UO_2_(L_NH_) and UO_2_(L_O_). Consequently, the bond strength of U–X interactions follows U–O ≈ U–NH > U–S. As widely accepted in the HSAB principle, the hardness of X moiety follows O > NH > S.^14,15^ Therefore, the trend of U–X bond strengths of UO_2_(L_X_) cannot be explained only by the HSAB principle, while the bond strength of U(1)–S(1) of UO_2_(L_S_) is certainly weaker than others. Note that all *R*_UX_ of UO_2_(L_X_) presented here are much smaller than those of noncovalent intermolecular interactions such as Cl⋯X and hydrogen bonds reported previously, where *R* = 0.98–0.80.^45–47^ Therefore, a coordination bond is certainly formed between U and X in each UO_2_(L_X_).

### Thermodynamic stability of UO_2_(L_X_) (X = NH, O, S)

**Fig. 3:**
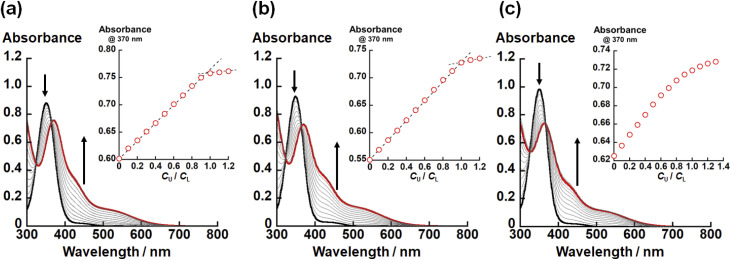
UV-vis absorption spectra of the ethanol solutions of (a) L_NH_^2−^, (b) L_O_^2−^ and (c) L_S_^2−^ at different total UO_2_^2+^ concentrations under the presence of 0.4 mM NEt_3_. Total concentration of L_X_^2−^: 0.1 mM. Black and red lines are spectra of *C*_U_/*C*_L_ = 0 and 1.0, respectively. Insets: plots of absorbance at 370 nm with an increase in *C*_U_/*C*_L_.

In the crystal structures of UO_2_(L_X_) (X = NH, O, S), the U–X bond strength depends on the difference in X. Therefore, there would also be some impact on the thermodynamic stability of UO_2_(L_X_). To confirm this issue, we investigated the complexation of UO_2_^2+^ and L_X_^2−^ in ethanol by spectrophotometric titration. Fig. 3 shows the UV-vis absorption spectra recorded at different total concentration ratios between UO_2_^2+^ and L_X_^2−^ represented by *C*_U_/*C*_L_. Note that these titration experiments were conducted under the presence of 0.4 mM NEt_3_ employed as a H^+^ scavenger after the formation of UO_2_(L_X_).

In all titration series shown in Fig. 3, the absorbance at 370 nm and 425 nm increased with an increase in *C*_U_/*C*_L_. Simultaneously, the absorption intensity at 350 nm decreased. The isosbestic points were clearly observed, indicating that the complexation equilibrium between UO_2_^2+^ and L_X_^2−^ only takes place in each system. As shown in the insets of Fig. 3(a) and (b), the absorbance at 370 nm tends to be saturated at *C*_U_/*C*_L_ = 1.0, indicating that UO_2_(L_NH_) and UO_2_(L_O_) are almost quantitatively formed. On the other hand, such a trend is equivocal for X = S (Fig. 3(c)), implying that the weaker coordination of L_S_^2−^.

To estimate the conditional stability constants (*β*_X_ = [UO_2_(L_X_)]/([UO_2_^2+^][L_X_^2−^])) of UO_2_(L_X_) (X = NH, O, S) in ethanol containing NEt_3_ (0.4 mM), the spectral series of Fig. 3 were analyzed by HypSpec program.^30^ As a result, log *β*_X_ of UO_2_(L_X_) for X = NH, O, and S are estimated to be 10 ± 1, 7.24 ± 0.02, and 5.2 ± 0.1, respectively. Since all the coordinating atoms except for X are common in the studied systems, the difference in log *β*_X_ observed here can be ascribed to the difference in affinity of X with UO_2_^2+^. As widely-accepted in the HSAB principle, the hardness of X atoms follows O > NH > S.^14,15^ However, log *β*_X_ of UO_2_(L_X_) decrease in the order of UO_2_(L_NH_) > UO_2_(L_O_) > UO_2_(L_S_), which is difficult to be rationalized only by the HSAB principle. To understand this trend, we focus on difference in basicity of X in L_X_^2−^. The p*K*_a_ values of protonated diethylamine ((CH_3_CH_2_)_2_NH_2_^+^), dimethyl ether ((CH_3_)_2_OH^+^), and dimethyl thioether ((CH_3_)_2_SH^+^) are 11.0 (ref. 48), −3.8 (ref. 48), and −5.4 (ref. 48), respectively, which is exactly in line with the order of log *β*_X_ of UO_2_(L_X_) described above. Therefore, the basicity of X atom would also provide some contribution to the thermodynamic stability of UO_2_(L_X_). At this moment, it is still too early to verify linear free energy relationship between log *β*_X_ and p*K*_a_.

To further elucidate the nature of U–X bonds in UO_2_(L_X_), we carried out DFT calculations of UO_2_(L_X_), followed by the natural bond orbital (NBO) analysis. The molecular structures of UO_2_(L_X_) were taken from those of UO_2_(L_X_) determined by X-ray crystallography, and were optimized with B3LYP method.^32^ The optimized structures of UO_2_(L_X_) are shown in Fig. S4,† and selected bond lengths are summarized in Table S2.† All the bond distances well agree with those determined crystallographically (Table 1). Table S3† summarizes natural charges and Wiberg bond indices (WBI)^17,18,40^ of center U and coordinating atoms in the optimized structures.

No significant differences were found in the natural charge on the axial and equatorial coordinating atoms except for X. Both N(3) in UO_2_(L_NH_) (−0.646) and O(5) in UO_2_(L_O_) (−0.565) have negative natural charges, indicating that the center U and X atoms interact electrostatically. In contrast, the natural charge of S(1) in UO_2_(L_S_) is positive (+0.326), implying that the electrostatic attraction between U and S is little expectable despite significant penetration between these atoms in UO_2_(L_S_) within the sum of van der Waals radii as described above. To provide a rationale for the U–S bonding interaction experimentally observed, bond orders of U–X interactions were estimated in terms of WBI. As a result, some covalency was detected in the U–S bond of UO_2_(L_S_) as pronounced by WBI = 0.471, which is significantly larger than those of the other U–X bonds (WBI = 0.277–0.345). Therefore, the bonding interaction between U and S of UO_2_(L_S_) is rather covalent, while it is somewhat weakened by the electrostatic repulsion between these positively charged atoms. The significant covalency of the U–S interaction compared with the electrostatic characters in U–NH and U–O would be a typical manifestation of the HSAB principle. In connection with this, N is usually considered to be softer than O, while the stability of UO_2_(L_NH_) is greater than UO_2_(L_O_) despite the hardness of UO_2_^2+^. The stronger basicity of NH provides an additional effect to strengthen the U–NH bond compared with that of U–O.

### Electrochemistry and spectroelectrochemistry of UO_2_(L_X_) (X = NH, O, S)

**Fig. 4:**
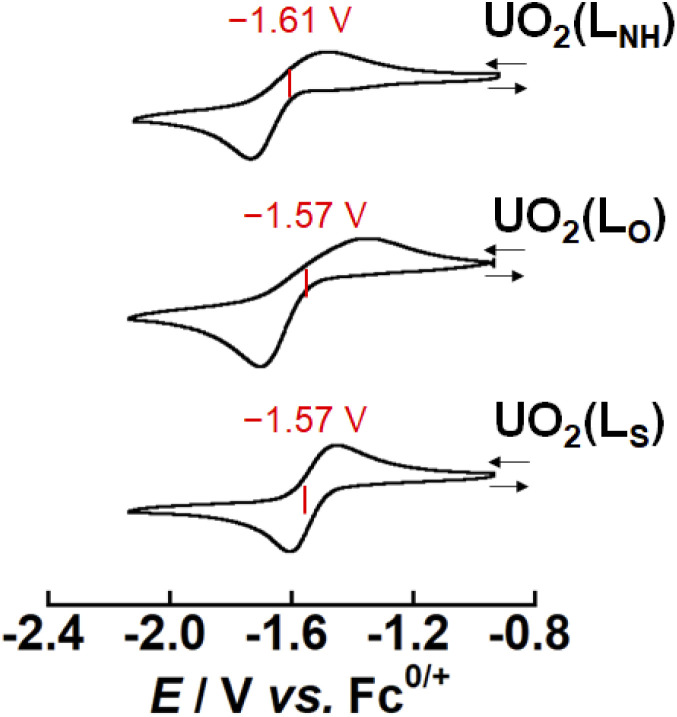
Cyclic voltammograms for the redox couples of UO_2_(L_X_) (X = NH, O, S) in DMSO at 295 K. Concentration of the complex was adjusted to 1 mM and tetra-*n*-butylammonium perchlorate (0.1 M) was used as a supporting electrolyte. Scan rates are 100 mV s^−1^.

**Fig. 5:**
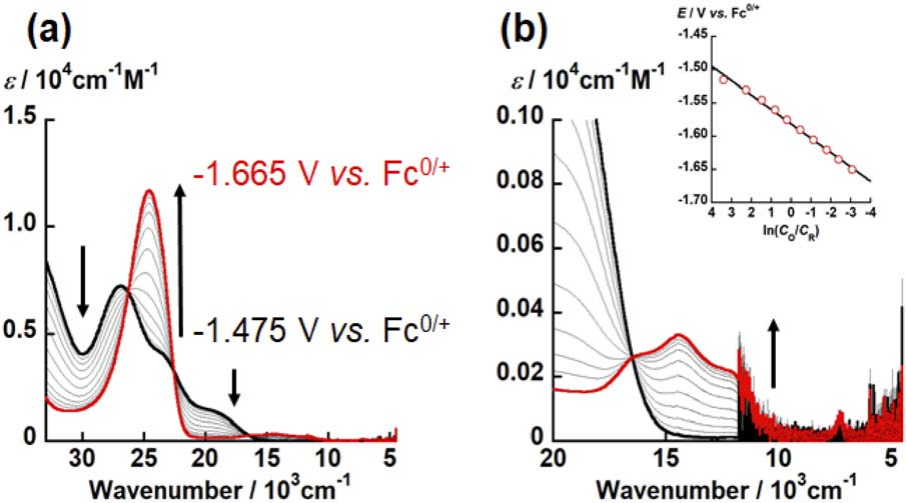
UV-vis-NIR spectral change of electrochemical reduction of UO_2_(L_O_) recorded at different applied potentials from −1.475 V to −1.665 V *vs.* Fc^0/+^ (potential step: 0.015 V) in DMSO with 0.1 M TBAP at 295 K. Black and red bold curves represent absorption spectra of UO_2_(L_O_) and [UO_2_(L_O_)]^−^, respectively. Wavenumber regions: (a) 33 333–4500 cm^−1^, (b) 20 000–4500 cm^−1^. Inset: Nernstian plot calculated from absorbance at 24 630 cm^−1^.

As mentioned above, X strongly affects the thermodynamic stability of UO_2_(L_X_). Recently, we have reported that the redox potential of UO_2_(R_1_,R_2_–^Me^saldien) (Fig. 1(a)) is significantly governed by substitution at R_1_ and R_2_ positions. Therefore, we expect that the difference in X may also vary the redox potentials of UO_2_(L_X_). To clarify this point, the electrochemical measurements of UO_2_(L_X_) in DMSO were carried out. Fig. 4 shows the obtained cyclic voltammograms of UO_2_(L_X_), where a couple of cathodic (*E*_pc_) and anodic peaks (*E*_pc_) has been observed. These redox waves are reproducible even in multiple scanned cyclic voltammograms recorded at the potential sweep rate (*ν*) of 100 mV s^−1^, indicating that the reduction product at *E*_pc_ undergoes no successive reactions, and is fully reoxidized to UO_2_(L_X_) at *E*_pa_ (Fig. S6†). The peak potential separation (*E*_pc_ − *E*_pa_) tends to increase (111–490 mV) with increasing *v* from 50 mV s^−1^ to 500 mV s^−1^ (Fig. S7 and Tables S4–S6†), implying that these redox systems of UO_2_(L_X_) are quasireversible. Even after careful survey of the DFT calculations described later, we, however, could not find any critical rationales for the differences in the electrochemical reversibility of these systems. Anyway, the peak potential separations (*E*_pa_ − *E*_pc_, see Table S6 in ESI†) of UO_2_(L_S_) were also much greater than the theoretical value of a reversible system (59 mV). Therefore, all the systems studied here are regarded to be electrochemically irreversible. Although we do not have unequivocal explanation for the above points at this moment, solvation structures around these uranyl complexes could be largely modified through the electron transfer. Note that all the redox reactions are chemically reversible as demonstrated by occurrence of the isosbestic points in the spectroelectrochemical experiments shown in Fig. 5, S8 and S9.† The diffusion coefficients (*D*_o_) of UO_2_(L_NH_), UO_2_(L_O_), and UO_2_(L_S_) in these systems at 295 K were estimated as 1.6 × 10^−6^, 1.8 × 10^−6^ and 8.1 × 10^−7^ cm^2^ s^−1^, respectively, where the redox reactions observed in Fig. 4 were assumed to be electrochemically irreversible.^49^ As summarized in Tables S4–S6,† the formal potential *E*°′ (=(*E*_pc_ + *E*_pa_)/2) of each UO_2_(L_X_) is around −1.60 V *vs.* Fc^0/+^ with regardless of *v*, and also seems not to be largely affected by X. The *E*°′ value of UO_2_(L_NH_) well agrees with that of its analogue, UO_2_(*t*Bu,MeO–^Me^saldien), we reported previously (−1.60 V *vs.* Fc^0/+^ in DMSO).^21,22^ Therefore, the coordinating L_X_^2−^ would not have strong contribution to the redox events of UO_2_(L_X_). From these results, we assume that the redox centers of all UO_2_(L_X_) are the UO_2_^2+^ moiety. However, cyclic voltammograms does not provide any detailed information about the reductant of UO_2_(L_X_). Hence, we carried out the spectroelectrochemical measurements and theoretical calculations to further understand the redox chemistry of UO_2_(L_X_).

To determine the electron stoichiometry (*n*) in the reduction of UO_2_(L_X_), the spectroelectrochemical measurements were performed. The UV-vis-NIR spectra of each system were recorded at different potentials (*E*). Fig. 5, S8 and S9† show the obtained spectral variations at X = O, NH, and S, respectively. As a general trend, the absorption bands of UO_2_(L_X_) around 30 000 and 20 000 cm^−1^ gradually decreased with decreasing *E*, while new absorption bands appeared around 25 000 and 15 000 cm^−1^. Moreover, isosbestic points were clearly observed, indicating that the redox equilibria of UO_2_(L_X_) only take place in the current potential ranges. Using the absorbance at 24 630 or 24 876 cm^−1^, the concentration ratio (*C*_O_/*C*_R_) between the oxidant (UO_2_(L_X_)) and its reductant at each *E* was calculated. The relationship between *C*_O_/*C*_R_ and *E* should follow the Nernstian equation, eqn (2).

2 *E* = *E*°′ + (*RT*/*nF*)ln(*C*_O_/*C*_R_)

where *E*°′, *R*, *T*, and *F* are the formal potential, the gas constant (8.314 J mol^−1^ K^−1^), the absolute temperature, and the Faraday constant (96 485 C mol^−1^), respectively. The slope and intercept of the linear relationship between *E* and ln(*C*_O_/*C*_R_) (insets of Fig. 5(b), S8(b) and S9(b)†) allow to determine *n* and *E*°′ of the redox reactions of UO_2_(L_X_). The estimated *n* values of UO_2_(L_X_) are close to unity (Table S7†), indicating that the reduction of UO_2_(L_X_) affords [UO_2_(L_X_)]^−^. The *E*°′ values estimated from the spectroelectrochemical measurements (Table S7†) agree with those observed in the CV measurements (Tables S4–S6†).

**Fig. 6:**
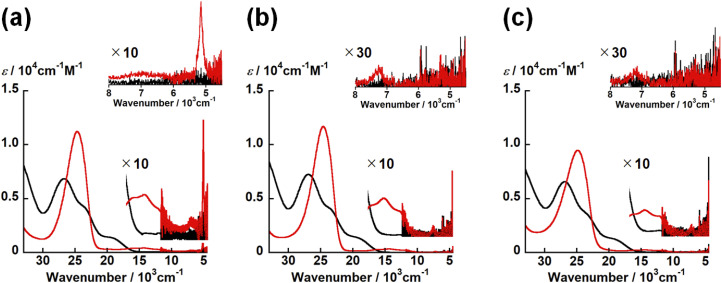
UV-vis-NIR spectrum of UO_2_(L_X_) (black) and one-electron reduced complexes [UO_2_(L_X_)]^−^ (red) in DMSO containing 0.1 M tetra-*n*-butylammonium perchlorate at 295 K. [UO_2_(L_NH_)]^−/0^ (a), [UO_2_(L_O_)]^−/0^ (b) and [UO_2_(L_S_)]^−/0^ (c). Inset: expended views of NIR region.

UV-vis-NIR spectra of UO_2_(L_X_) and [UO_2_(L_X_)]^−^ in DMSO were summarized in Fig. 6. The spectral features of all UO_2_(L_X_) are quite similar to each other. All UO_2_(L_X_) showed characteristic bands around 28 000 and 24 000 cm^−1^. These absorption bands were also observed in UO_2_^2+^ complexes with Schiff base ligands, and can be assigned to the π–π* transition bands of Schiff base ligands.^21,22^ Therefore, the difference in X leads to no significant differences in the electronic structures of UO_2_(L_X_).

Even after the reduction, [UO_2_(L_X_)]^−^ with different X commonly have the intense bands at around 25 000 cm^−1^ with *ε* ∼ 10^4^ M^−1^ cm^−1^ and weak bands at 16 400, 14 500, 12 200 and 7200 cm^−1^ with *ε* ∼ 10^2^ M^−1^ cm^−1^ (Fig. 6). Note that [UO_2_(L_NH_)]^−^ has a characteristic band at 5200 cm^−1^, although this absorption is not clearly observed in [UO_2_(L_O_)]^−^ and [UO_2_(L_S_)]^−^ (Fig. 6). The absorption bands at around 25 000, 16 400, 14 500, 12 200 and 7200 cm^−1^ are generally observed in U^V^O_2_^+^ complexes with Schiff base ligands as reported previously.^21,22^ The intense absorption at 25 000 cm^−1^ is assigned to a π–π* transition in the Schiff base ligands and/or a ligand-to-metal charge transfer (LMCT).^21,22^ In accordance with TD-DFT calculation,^21,22^ the absorption band at 16 400 cm^−1^ is attributable to a metal-to-ligand charge transfer (MLCT) from a 5f*δ*_u_ orbital of the U^5+^ center to the π* orbital of the coordinating ligand. Finally, those at 14 500, 12 200 and 7200 cm^−1^ are ascribed to the f–f transitions arising from the 5f^1^ electron configuration of U^5+^.^21,22,35^ [UO_2_(L_NH_)]^−^ only exhibited the absorption band at 5200 cm^−1^ attributable to another f–f transition,^21,22,35^ while this is not the case for the others studied here. As a matter of fact, this transition is not always clearly observable as we reported previously.^22^ To theoretically support occurrence of U^V^O_2_^+^ in each [UO_2_(L_X_)]^−^, we further performed DFT calculations of [UO_2_(L_X_)]^−^.

Initially, the molecular structures of [UO_2_(L_X_)]^−^ in DMSO were taken from those of UO_2_(L_X_) determined by the X-ray crystal structure and were optimized after addition of a single negative charge and doublet spin degeneracy to assume the one-electron reduction. The optimized structures of [UO_2_(L_X_)]^−^ were shown in Fig. S10† and the selected structural parameters are summarized in Table S2.†

**Fig. 7:**
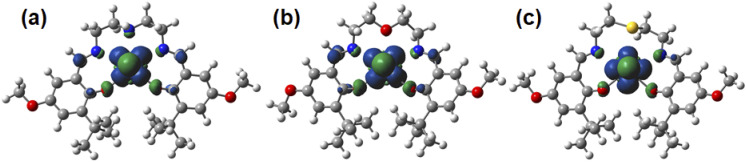
Spin-density plots of [UO_2_(L_NH_)]^−^ (a), [UO_2_(L_O_)]^−^ (b), [UO_2_(L_S_)]^−^ (c). Spin density values of U atom are 1.09 for [UO_2_(L_NH_)]^−^, 1.09 for [UO_2_(L_O_)]^−^, 1.12 for [UO_2_(L_S_)]^−^.

The U≡O_ax_ bond lengths of [UO_2_(L_X_)]^−^ are 1.86 Å, which are *ca.* 0.08 Å longer than those of the corresponding UO_2_(L_X_) determined by SCXRD (Table 1). These U≡O_ax_ bond lengths are very similar to those of the U^V^O_2_^+^ complexes with bis(phenolate) ligands (1.851(7)–1.868(8) Å).^50^ The U–O bond lengths between U atom and phenolic O of [UO_2_(L_X_)]^−^ are *ca.* 0.15 Å longer than those of the corresponding UO_2_(L_X_) (Table 1). These U–O bond elongations indicate that these bond strengths are weakened by a decrease in the positive charge of U through the reduction from U^VI^O_2_^2+^ to U^V^O_2_^+^. Actually, an unpaired electron of [UO_2_(L_X_)]^−^ is exclusively localized in the center U as shown in the Mulliken spin density surfaces (Fig. 7), clearly indicating that these reduced complexes are of U^V^O_2_^+^ regardless of difference in X. Consequently, the X moiety does not largely affect the redox chemistry of [UO_2_(L_X_)]^−/0^.

## Conclusions

In this study, UO_2_(*t*Bu,MeO–saldien–X) (UO_2_(L_X_); X = NH, O, S) were synthesized and structurally characterized to discuss impacts of the heteroatoms (X) to the coordination chemistry of UO_2_^2+^. The crystal structures of UO_2_(L_X_) showed the five-coordinated UO_2_^2+^ with L_X_^2−^ in the equatorial plane. The U≡O_ax_ bond length of UO_2_^2+^ and the bond length between U and phenolic O are not affected by the difference in X. On the other hand, the U–X bond length increases in the order of UO_2_(L_O_) < UO_2_(L_NH_) < UO_2_(L_S_). After taking into account the differences in the atomic size of X, the normalized U–X bond strength in UO_2_(L_X_) was found to follow U–O ≈ U–NH > U–S. While the U–O and U–NH bond strengths are similar to each other, the weaker U–S interaction can be explained by the HSAB principle. The logarithmic conditional stability constant (log *β*_X_) of UO_2_(L_X_) in ethanol containing 0.4 mM NEt_3_ decreases in the order of UO_2_(L_NH_) (log *β*_NH_ = 10) > UO_2_(L_O_) (log *β*_O_ = 7.24) > UO_2_(L_S_) (log *β*_S_ = 5.2). This trend cannot be explained only by the HSAB principle, but rather follows the order of basicity of X. The theoretical calculations of UO_2_(L_X_) suggested that the ionic character of U–X bonds decreases in the order of U–NH > U–O > U–S. In contrast, the covalency increases as U–O < U–NH < U–S. No significant differences were found in the electrochemistry of UO_2_(L_X_) with different X in terms of *E*°′ and U-centered redox reaction. As demonstrated in this work, a UO_2_^2+^-ligand bond strength does not always follow the HSAB principle, but is also affected by other factors such as Lewis basicity and balance between ionic and covalent interactions of donating atoms to the center metal. These points should be more carefully considered to design molecular structures of ligands suitable for hydrometallurgical separations of metal ions of interest.

## Author contributions

T. T. devised the main conceptual ideas, carried out all experiments, and wrote the manuscript in consultation with K. T. K. T. supervised this project, discussed all experimental results with T. T., and edited the manuscript. All authors have given approval to the final version of the manuscript.

## Conflicts of interest

There are no conflicts to declare.

## Supplementary Material

RA-012-D2RA04639C-s001

RA-012-D2RA04639C-s002
