# Supplementary material for: Effects of coordinating heteroatoms on molecular structure, thermodynamic stability and redox behavior of uranyl(vi) complexes with pentadentate Schiff-base ligands
Source: RSC Adv. 2022 Aug 26;12(37):24260–8. doi: 10.1039/d2ra04639c (PMC9413499; doi:10.1039/d2ra04639c)
Supplement: RA-012-D2RA04639C-s001 [file RA-012-D2RA04639C-s001.pdf]

Electronic Supplementary Material (ESI) for RSC Advances.
This journal is © The Royal Society of Chemistry 2022

Electronic Supplementary Information (ESI)

Effects of coordinating heteroatoms on molecular structure, thermodynamic stability and redox behavior of uranyl(VI) complexes with pentadentate Schiff-base ligands.

Tomoyuki Takeyama,*[,a] Koichiro Takao*[,a]

AUTHOR ADDRESS.

[a] Laboratory for Zero-Carbon Energy, Institute of Innovative Research, Tokyo Institute of Technology 2-12-1 N1-32, O-okayama, Meguro-ku, Tokyo 152-8550, Japan

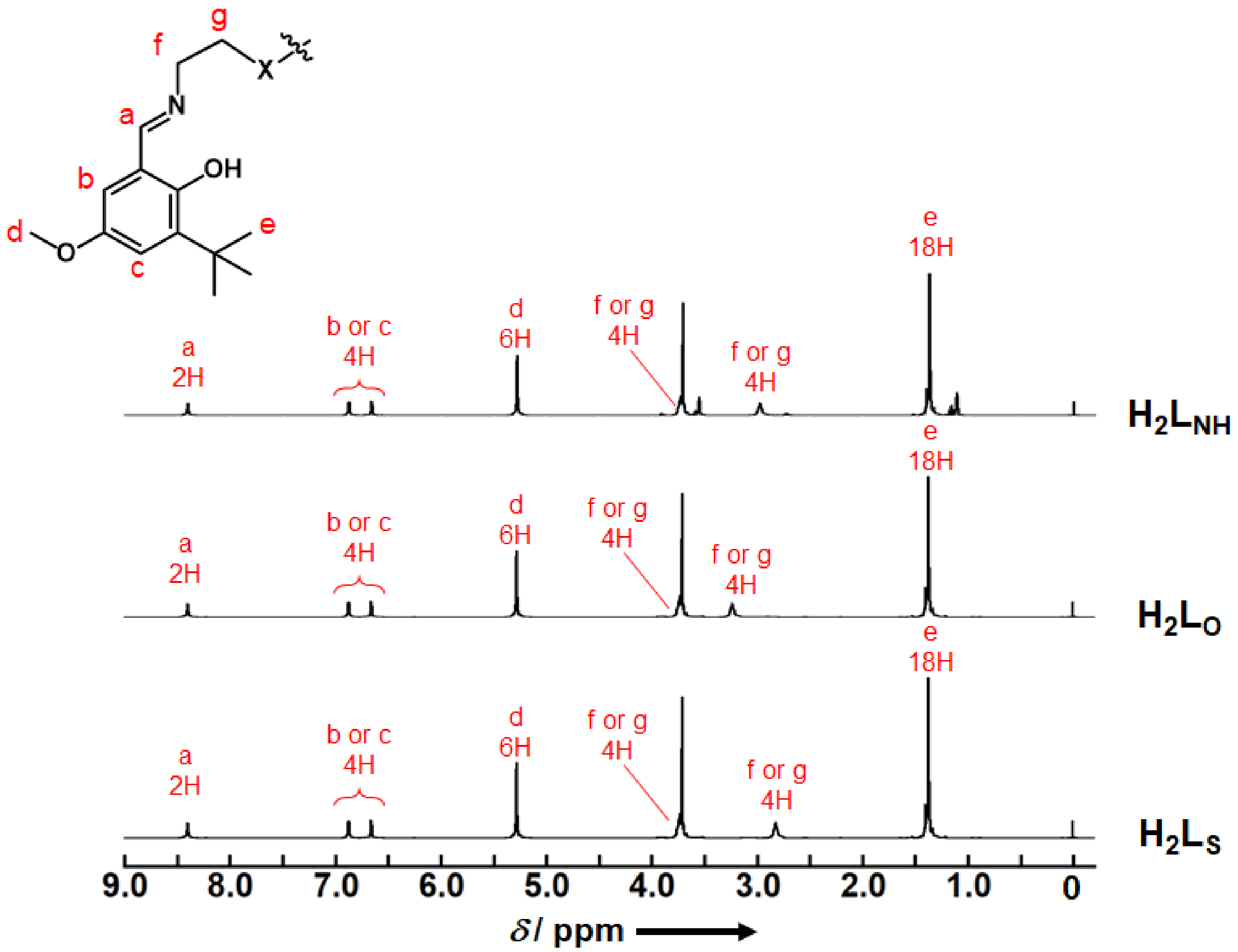

Figure S1. ^{1}H NMR spectra of H_2L_{NH} (a), H_2L_O (b) and H_2L_S (c) in CD_3CD_2OD.

Table S1. Crystallographic data for of $\mathbf{UO_2(L_{NH})}\cdot(CH_2Cl_2)$, $\mathbf{UO_2(L_O)}\cdot(C_5H_5N)$ and $\mathbf{UO_2(L_S)}\cdot(CH_2Cl_2)$.

	$\mathbf{UO_2(L_{NH})}\cdot(CH_2Cl_2)$	$\mathbf{UO_2(L_O)}\cdot(C_5H_5N)$	$\mathbf{UO_2(L_S)}\cdot(CH_2Cl_2)$
Formula	$C_{29}H_{41}N_3O_6U_1Cl_2$	$C_{33}H_{43}N_3O_7U_1$	$C_{29}H_{40}N_2O_6U_1S_1Cl_2$
Formula weight	836.58	831.73	853.62
Colour	red	red	red
Crystal size / mm	0.26×0.16×0.06	0.18×0.17×0.08	0.66×0.22×0.05
Crystal system	monoclinic	triclinic	monoclinic
Space group	$P2_1/c$	$P-1$	$P2_1/c$
a(Å)	9.9547(2)	10.19370(10)	16.3889(14)
b(Å)	29.1269(5)	13.0886(2)	18.8968(9)
c(Å)	11.6812(2)	13.2854(2)	10.3557(6)
α(°)	-	75.1030(10)	-
β(°)	106.798(2)	80.7980(10)	98.602(7)
γ(°)	-	71.7900(10)	-
V(Å^3)	3242.44(11)	1621.06(4)	3171.1(4)
Z	4	2	4
T (K)	123	123	123
μ (mm^{-1})	5.214	5.057	5.396
F(000)	1640.00	820.00	1672.00
D_{culc}(g/cm^3)	1.714	1.704	1.788
$2\Theta_{max}$	30.960	30.5830	30.890
No. reflections obsd.	9910	9686	9534
No. reflections used.	7869	8730	5994
No. variables	396	375	336
R_1[a] ($I > 2\sigma(I)$)	0.0333	0.0287	0.0662
wR_2 (all)[b]	0.0624	0.0673	0.1747

[a]$R_1 = \Sigma||F_o|-|F_c|| / \Sigma|F_o|$ for $I > 2\sigma(I)$ data.
[b] wR_2 (all) = $\{\Sigma\omega(|F_o|-|F_c|)2 / \Sigma\omega F_o^2\}^{1/2}$; $\omega = 1/\sigma^2(F_o) = \{\sigma^2_c(F_o) + p^2/4\cdot F_o^2\}^{-1}$

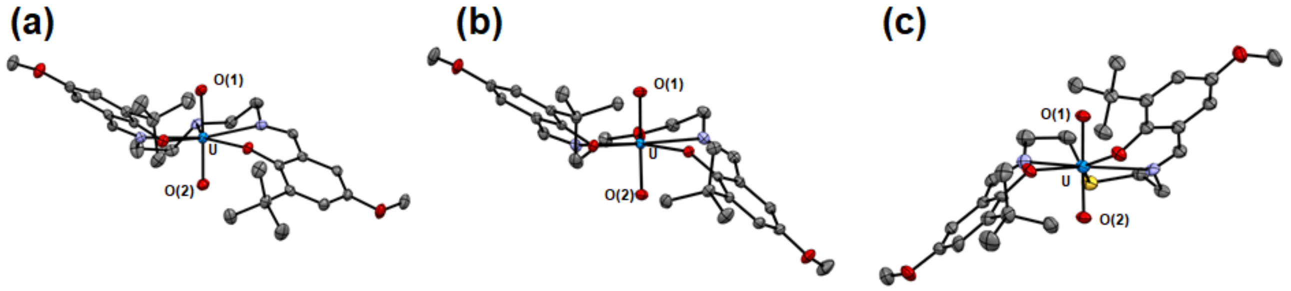

Figure S2. ORTEP view of **$UO_2(L_{NH})$** (a), **$UO_2(L_O)$** (b) and **$UO_2(L_S)$** (c). Ellipsoids are at 50% probability. Hydrogen atoms were omitted by clarify.

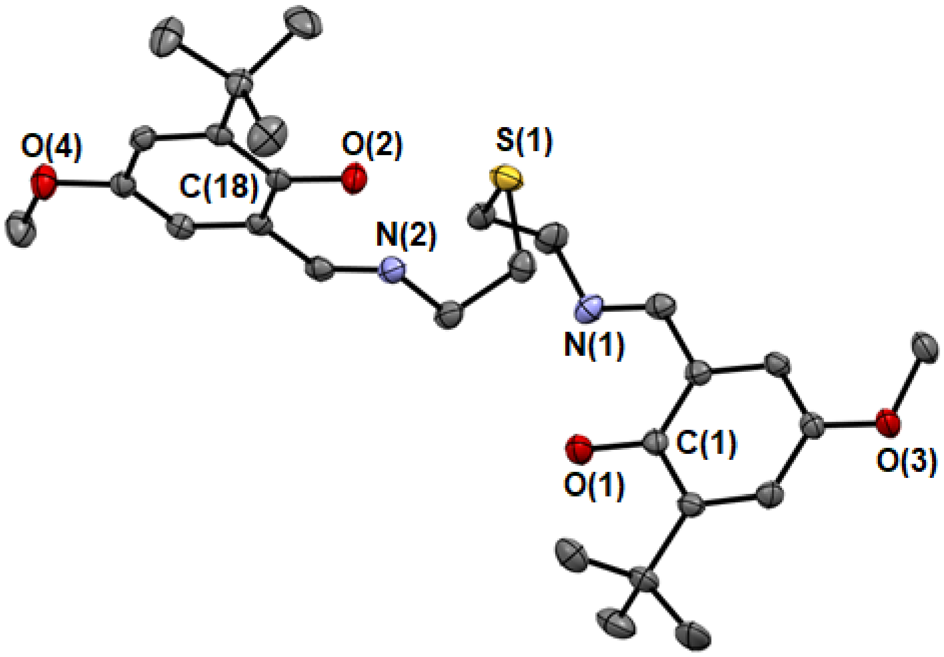

Figure S3. ORTEP view of H_2L_S. Ellipsoids are at 50% probability. Hydrogen atoms were omitted by clarify. *F*w = 500.68, 0.57 × 0.32 × 0.08 mm^3, triclinic, *P*−1, *a* = 9.9818(8) Å, *b* = 11.6754(8) Å, *c* = 13.1840(8) Å, α = 99.572(6)°, β = 109.064(7)°, γ = 101.528(6)°, *V* = 1377.72(18) Å^3, *Z* = 2, *T* = 133 K, D_{calcd} = 1.207 g/cm^3, μ(Mo *K*α) = 0.152 mm^{-1}, GOF = 1.010, R_1 ($I > 2\sigma$) = 0.0520, wR_2 (all) = 0.1282.

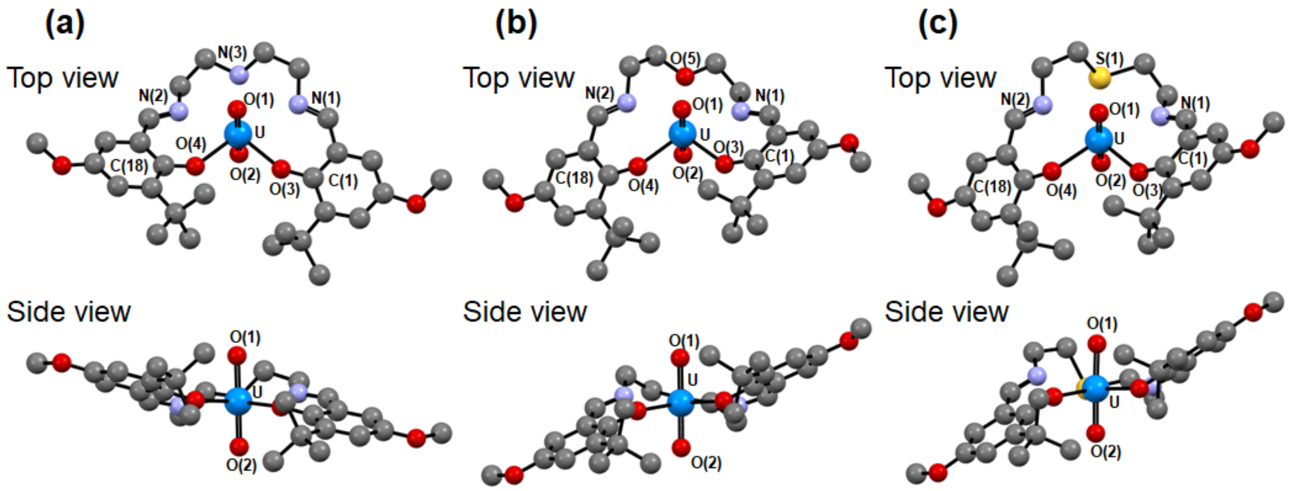

Figure S4. The optimized structures of **$UO_2(L_{NH})$** (a), **$UO_2(L_O)$** (b) and **$UO_2(L_S)$** (c).

Table S2. The selected bond lengths of calculated structures of **$UO_2(L_X)$** and [**$UO_2(L_X)$**]$^-$ (X = NH, O, S).

	**$UO_2(L_{NH})$**	[**$UO_2(L_{NH})$**]$^-$	**$UO_2(L_O)$**	[**$UO_2(L_O)$**]$^-$	**$UO_2(L_S)$**	[**$UO_2(L_S)$**]$^-$
U-O(1)	1.799	1.859	1.798	1.858	1.796	1.855
U-O(2)	1.799	1.858	1.798	1.859	1.796	1.853
U-O(3)	2.271	2.407	2.262	2.402	2.265	2.385
U-O(4)	2.279	2.407	2.274	2.384	2.277	2.398
U-N(1)	2.665	2.705	2.595	2.660	2.634	2.731
U-N(2)	2.624	2.732	2.584	2.668	2.636	2.734
U-X	2.648	2.692	2.622	2.689	3.052	3.121
C(1)-O(3)	1.319	1.299	1.323	1.300	1.322	1.302
C(18)-O(4)	1.320	1.298	1.319	1.303	1.322	1.302

Table S3. Selected Natural Charges and Wiberg Bond Indices in **$UO_2(L_X)$** (X = NH, O, S).

	**$UO_2(L_{NH})$**	**$UO_2(L_O)$**	**$UO_2(L_S)$**
Natural Charge			
U(1)	1.448	1.449	1.359
O(1)	−0.516	−0.514	−0.519
O(2)	−0.516	−0.515	−0.522
O(3)	−0.657	−0.658	−0.662
O(4)	−0.659	−0.658	−0.662
N(1)	−0.495	−0.486	−0.495
N(2)	−0.486	−0.482	−0.501
X	−0.646	−0.565	0.326
Wiberg Bond Index			
U(1)–O(1)	2.167	2.169	2.164
U(1)–O(2)	2.170	2.168	2.160
U(1)–O(3)	0.730	0.752	0.726
U(1)–O(4)	0.738	0.731	0.737
U(1)–N(1)	0.376	0.399	0.390
U(1)–N(2)	0.390	0.408	0.386
U(1)–X	0.345	0.277	0.471

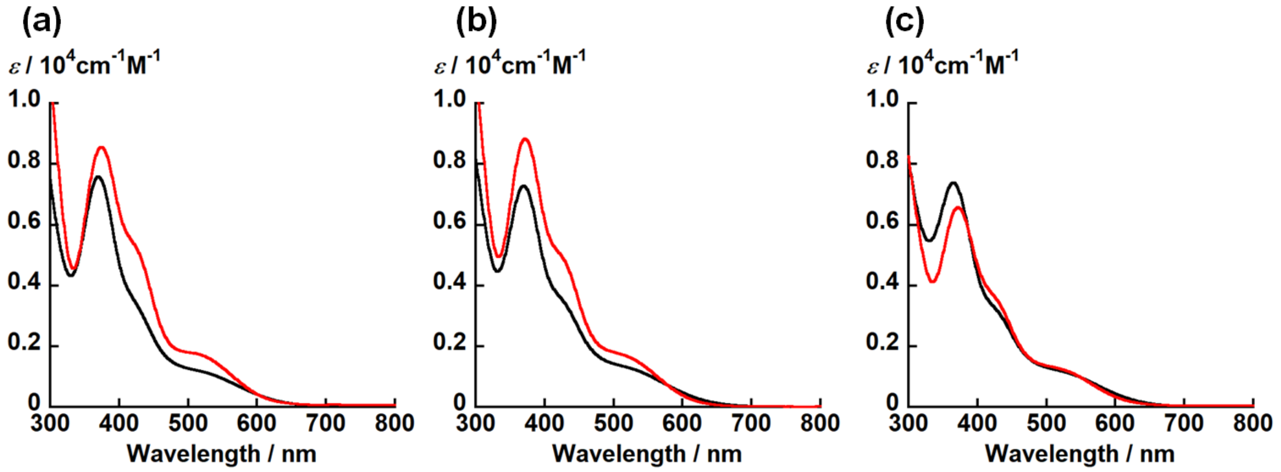

Figure S5. UV-vis absorption spectra of **UO_2(L_{NH})** (a), **UO_2(L_O)** (b), **UO_2(L_S)** (c) in ethanol containing NEt_3 (black) and DMSO (red) at 295 K.

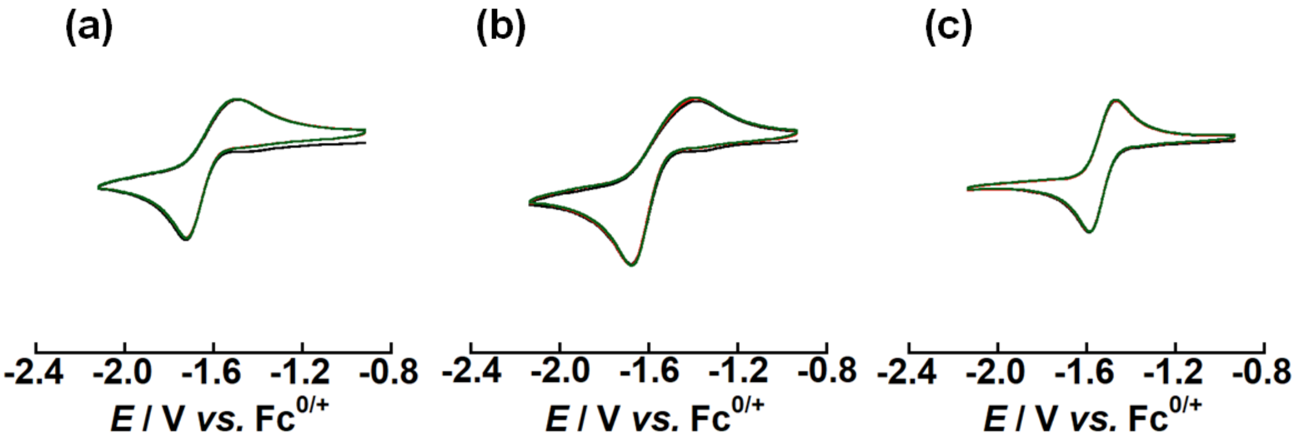

Figure S6. The multiple scanned cyclic voltammograms for the redox couples of **$UO_2(L_{NH})$** (a), **$UO_2(L_O)$** (b), **$UO_2(L_S)$** (c) in DMSO at 295 K. Concentration of the complex was adjusted to 1 mM and tetra-*n*-butylammonium perchlorate (0.1 M) was used as a supporting electrolyte. Potentials in the figures show the relative values to that of the $Fc^{0/+}$ redox couple. Scan rates are 50 $mV{\cdot}s^{-1}$. First, second and third scanned cyclic voltammograms are represented as black, red and green lines, respectively.

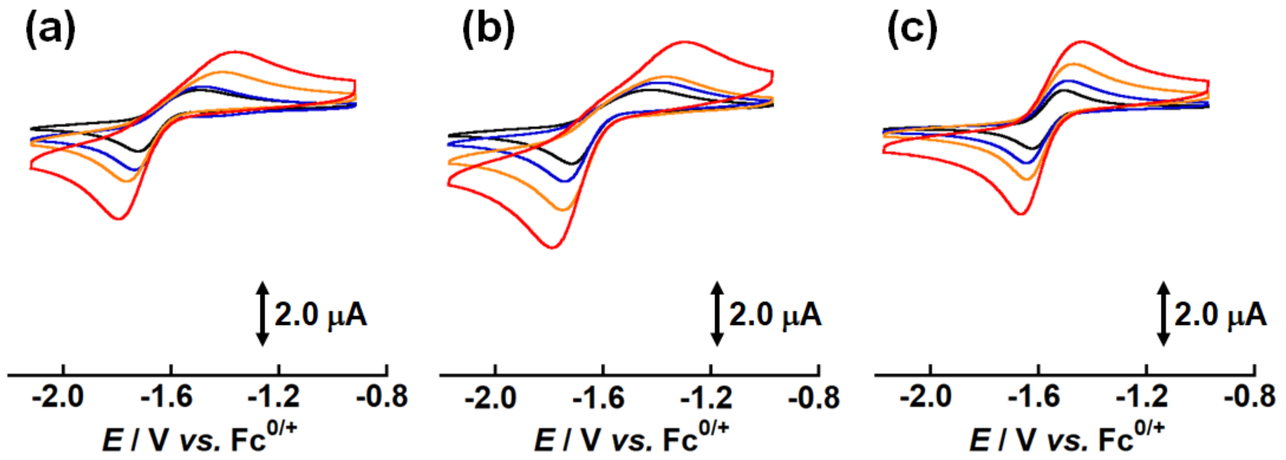

Figure S7. Cyclic voltammograms for the redox couples of **$UO_2(L_{NH})$** (a), **$UO_2(L_O)$** (b), **$UO_2(L_S)$** (c) in DMSO at 295 K. Concentration of the complex was adjusted to 1 mM and tetra-*n*-butylammonium perchlorate (0.1 M) was used as a supporting electrolyte. Potentials in the figures show the relative values to that of the $Fc^{0/+}$ redox couple. Scan rates are 50 mV·s^{-1} (black), 100 mV·s^{-1} (blue), 200 mV·s^{-1} (orange) and 500 mV·s^{-1} (red).

Table S4. The redox potentials of [$\mathbf{UO_2(L_{NH})}$]$^{–/0}$ systems in DMSO containing 0.1 M *tetra*-*n*-butylammonium perchlorate at 295 K (vs. $Fc^{0/+}$).

Scan rate	E_{pc} / V	E_{pa} / V	$E^{o'}$ / V	$E_{pa} − E_{pc}$ / V
50 $mV·s^{–1}$	–1.722	–1.496	–1.61	0.226
100 $mV·s^{–1}$	–1.737	–1.484	–1.61	0.253
200 $mV·s^{–1}$	–1.764	–1.414	–1.59	0.350
500 $mV·s^{–1}$	–1.796	–1.364	–1.58	0.432

Table S5. The redox potentials of [$\mathbf{UO_2(L_{O})}$]$^{–/0}$ systems in DMSO containing 0.1 M *tetra*-*n*-butylammonium perchlorate at 295 K (vs. $Fc^{0/+}$).

Scan rate	E_{pc} / V	E_{pa} / V	$E^{o'}$ / V	$E_{pa} − E_{pc}$ / V
50 $mV·s^{–1}$	–1.716	–1.436	–1.58	0.280
100 $mV·s^{–1}$	–1.745	–1.395	–1.57	0.350
200 $mV·s^{–1}$	–1.751	–1.372	–1.56	0.379
500 $mV·s^{–1}$	–1.792	–1.302	–1.55	0.490

Table S6. The redox potentials of [$\mathbf{UO_2(L_{S})}$]$^{–/0}$ systems in DMSO containing 0.1 M *tetra*-*n*-butylammonium perchlorate at 295 K (vs. $Fc^{0/+}$).

Scan rate	E_{pc} / V	E_{pa} / V	$E^{o'}$ / V	$E_{pa} − E_{pc}$ / V
50 $mV·s^{–1}$	–1.623	–1.512	–1.57	0.111
100 $mV·s^{–1}$	–1.646	–1.488	–1.57	0.158
200 $mV·s^{–1}$	–1.646	–1.477	–1.56	0.169
500 $mV·s^{–1}$	–1.663	–1.448	–1.56	0.215

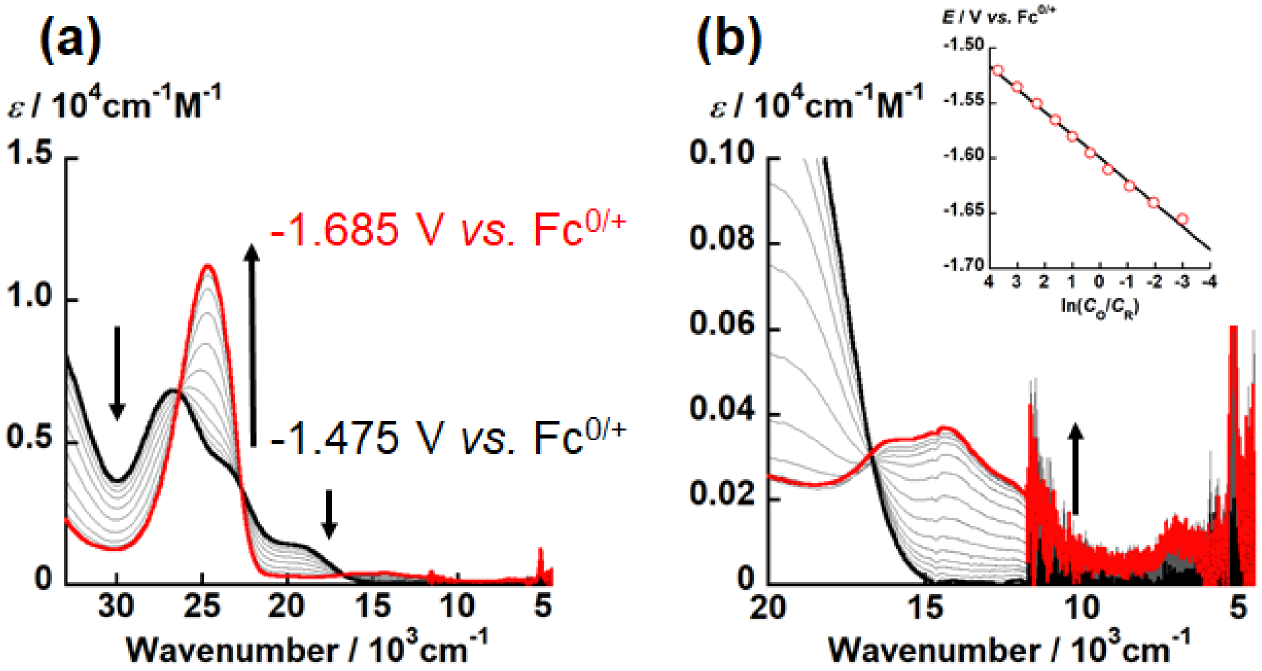

Figure S8. UV-vis-NIR spectral change of electrochemical reduction of **UO_2(L$_{NH}$)** recorded at different applied potentials from −1.475 V to −1.685 V vs. $Fc^{0/+}$ (potential step: 0.015 V) in DMSO with 0.1 M TBAP at 295 K. Black and red bold curves represent absorption spectra of **UO_2(L$_{NH}$)** and [**UO_2(L$_{NH}$)**]$^{-}$, respectively. Wavenumber regions: (a) 33333−4500 cm^{-1}, (b) 20000−4500 cm^{-1}. Inset: Nernstian plot calculated from absorbance at 24876 cm^{-1}.

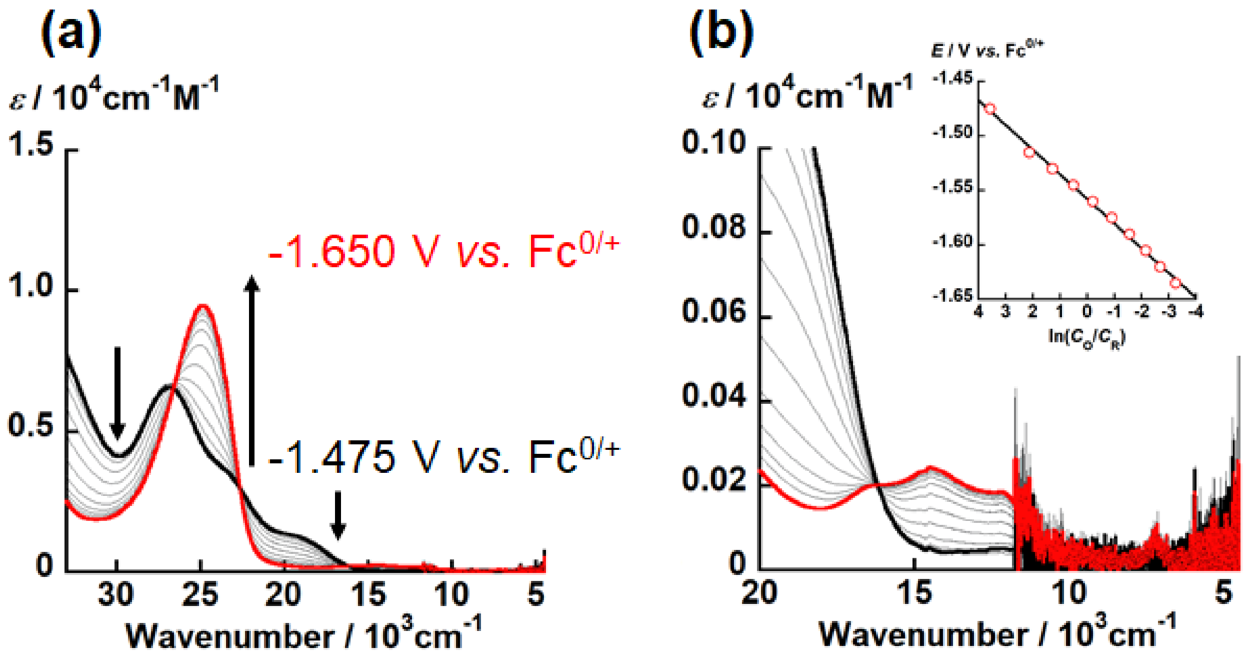

Figure S9. UV-vis-NIR spectral change of electrochemical reduction of **UO_2(Ls)** recorded at different applied potentials from −1.475 V to −1.650 V vs. $Fc^{0/+}$ (potential step: 0.015 V) in DMSO with 0.1 M TBAP at 295 K. Black and red bold curves represent absorption spectra of **UO_2(Ls)** and **[UO_2(Ls)]**$^-$, respectively. Wavenumber regions: (a) 33333−4500 cm^{-1}, (b) 20000−4500 cm^{-1}. Inset: Nernstian plot calculated from absorbance at 24876 cm^{-1}.

Table S7. The *n* and $E^{\circ\prime}$ values of **[UO_2(L_X)]**$^{-/0}$ (X = NH, O, S) estimated by spectroelectrochemical measurements.

	n	$E^{\circ\prime}$ / V (vs. $Fc^{0/+}$)
**[UO_2(L_{NH})]**$^{-/0}$	1.21(3)	−1.600(1)
**[UO_2(L_O)]**$^{-/0}$	1.18(2)	−1.581(1)
**[UO_2(L_S)]**$^{-/0}$	1.13(2)	−1.558(1)

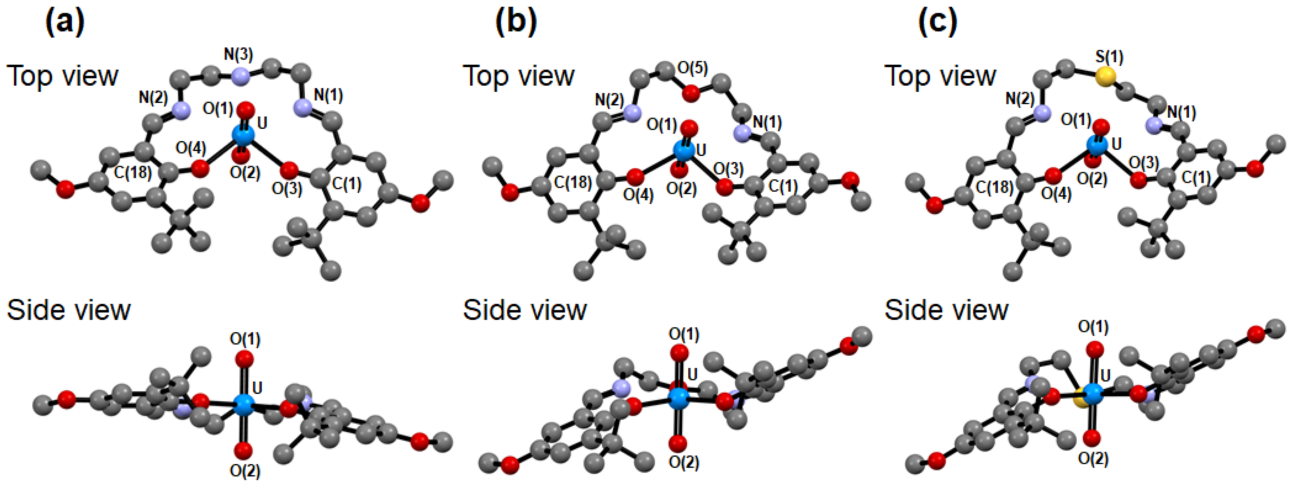

Figure S10. The optimized structures of [$\mathbf{UO_2(L_{NH})}$]$^-$ (a), [$\mathbf{UO_2(L_O)}$]$^-$ (b) and [$\mathbf{UO_2(L_S)}$]$^-$ (c).

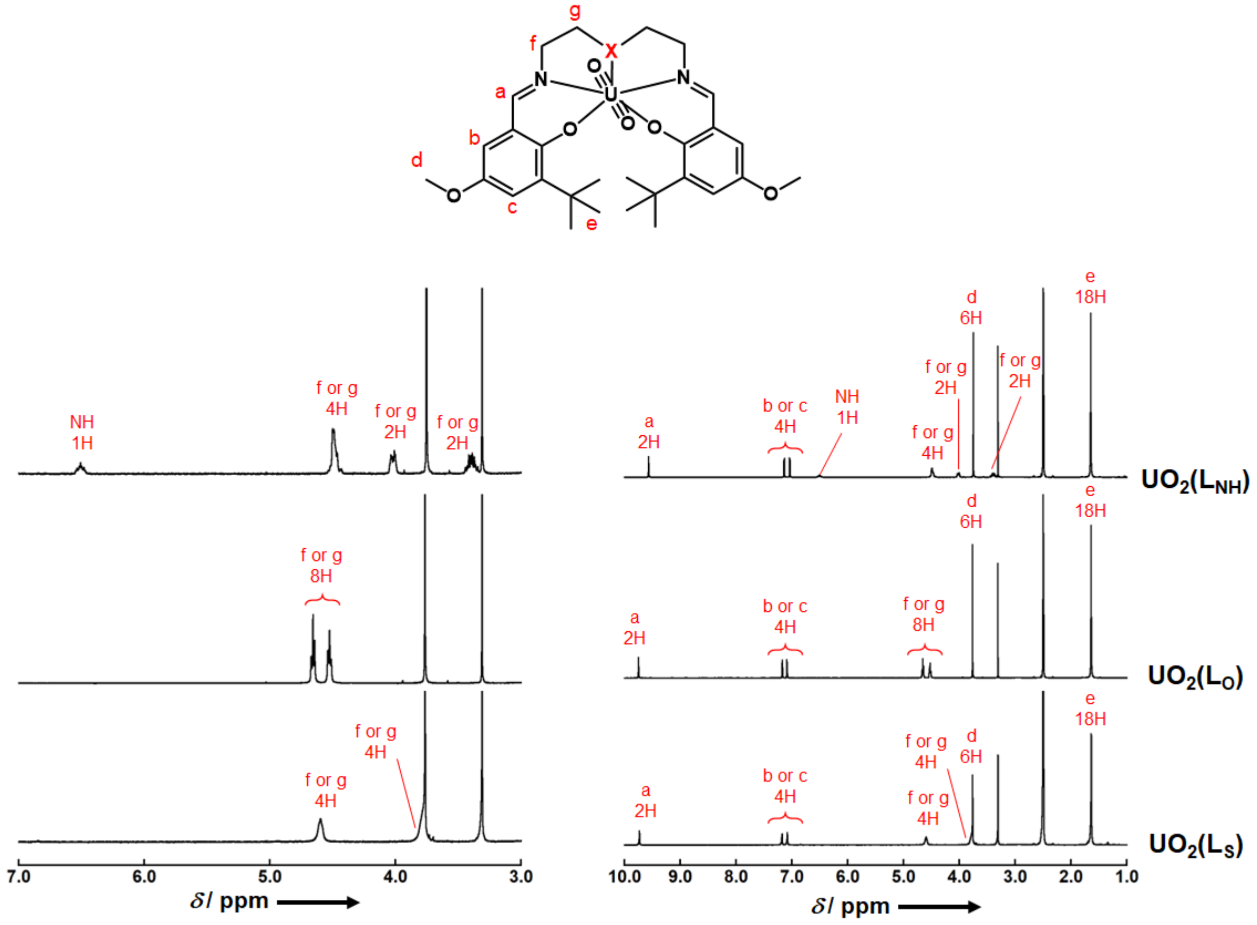

Figure S11. ^{1}H NMR spectra of $\mathbf{UO_2(L_{NH})}$, $\mathbf{UO_2(L_O)}$, and $\mathbf{UO_2(L_S)}$ in DMSO-d_6 at 295 K.
